# Comparative evaluation of a folkloric plant extract in a rat partial nephrectomy model

**DOI:** 10.1590/acb411026

**Published:** 2026-03-23

**Authors:** Ismail Ulus, Bulent Erol, Meftun Culpan, Sıdıka Seyma Ozkanli, Volkan Hanci, Turhan Caskurlu

**Affiliations:** 1 Bagcilar Training and Research Hospital – Department of Urology – Istanbul – Turkey.; 2 Bahceci Clinic – Department of Urology – Istanbul – Turkey.; 3 Memorial Hospital – Department of Urology – Istanbul – Turkey.; 4 Istanbul Medeniyet University – Faculty of Medicine – Department of Pathology – Istanbul – Turkey.; 5 Dokuz Eylül University – Faculty of Medicine – Department of Anesthesiology and Reanimation – Izmir – Turkey.

**Keywords:** Hemostatics, Nephrectomy, Models, Animal

## Abstract

**Purpose:**

To evaluate the efficacy and safety of Ankaferd in comparison with Arista and Surgiflo in a rat zero-ischemia partial nephrectomy model.

**Methods:**

A total of 28 Wistar albino rats were randomly assigned to four groups: control, Ankaferd, Arista, and Surgiflo. During partial nephrectomy, both the amount of bleeding and bleeding time were recorded. Intra-abdominal adhesions were evaluated on postoperative day 10. Nephrectomy specimens were harvested for histopathological assessment of tubular and glomerular necrosis, as well as for CD142 immunostaining.

**Results:**

Ankaferd demonstrated superior performance in terms of bleeding amount and bleeding time compared with both Arista and Surgiflo, although the differences did not reach statistical significance. Intra-abdominal adhesion scores were higher in the Ankaferd group than in the Arista and Surgiflo groups, with a statistically significant difference compared with the control group (*p* = 0.01). Histopathological analysis revealed a significantly higher glomerular necrosis score in the Ankaferd group compared with the control group (*p* = 0.043), while no significant differences were observed for tubular necrosis or CD142 immunohistochemical evaluation among the study groups.

**Conclusion:**

Ankaferd achieved more effective and rapid hemostasis than Arista and Surgiflo, but its higher adhesion scores limit its reliability for intra-abdominal use.

## Introduction

Partial nephrectomy is currently considered the gold standard treatment for localized renal tumors, offering comparable oncological outcomes to radical nephrectomy while significantly reducing the risk of chronic kidney disease and improving overall survival^1^. Nephron-sparing surgery employing a zero-ischemia technique is considered superior, as long-term renal function is primarily influenced by warm ischemia time and the volume of renal parenchyma preserved^2^. Various hemostatic agents have been investigated to enhance intraoperative hemostasis during nephron-sparing surgery, given the limited efficacy of conventional techniques such as pressure, ligature, cautery, and the use of various energy-based modalities.

Although numerous hemostatic agents with varying mechanisms of action have been developed, there remains no standardized protocol for selecting specific agents across different types of surgical procedures^3^. To meet this need, these agents should be evaluated for both efficacy and biocompatibility, which must be demonstrated through comparative animal studies prior to clinical trials.

Topical hemostatic agents are mainly categorized in three groups as matrix agents, biological agents, and synthetic agents^4^. Ankaferd (AND İlaç) is a folkloric medicinal plant extract composed of a mixture of five different plants—*Urtica dioica, Vitis vinifera, Glycyrrhiza glabra, Alpinia officinarum*, and *Thymus vulgaris*—, which have effects on endothelium, blood cells, and vascular dynamics^5^. It exerts hemostatic activity by forming a protein network that facilitates erythrocyte aggregation and erythrocyte-protein interactions, independently of the classical coagulation cascade.

In this study, we compared Ankaferd with the matrix agent Arista (Davol), a microporous polysaccharide sphere, and the biological agent Surgiflo (Ethicon), a flowable gelatin matrix, in terms of hemostatic efficacy and safety using a rat model of zero-ischemia partial nephrectomy. Additionally, immunohistochemical analysis of Tissue factor (CD 142), a membrane-bound glycoprotein that functions as a receptor for coagulation factor VII/VIIa, was performed. It is expressed on the surface of fibroblasts and smooth muscle cells lining blood vessels, as well as on cells surrounding the microvascular system, and its expression is upregulated during inflammatory and immune responses, indicating tissue damage^6^.

## Methods

This study was designed and reported in accordance with the Animal Research: Reporting of In Vivo Experiments (ARRIVE) guidelines and was approved by the local Animal Ethics Committee and the Research Council of Marmara University, Istanbul (Approval No: 53.2013.mar)^7^.

A total of 28 healthy, female Wistar albino rats weighing 350–420 g were obtained from Marmara University Experimental Animals Research and Implementation Center. The animals were housed in a climate-controlled facility (24 ± 1°C) under a 12-hour light/12-hour dark cycle. Food and water were available *ad libitum*. Randomization into four groups (Ankaferd, Arista, Surgiflo, and control) was performed using computer-generated random numbers, with each group consisting of seven animals.

**Figure 1:**
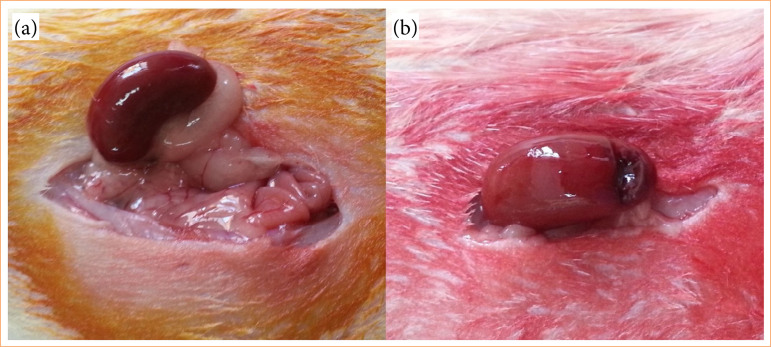
Zero-ischemia partial nephrectomy steps. (a) Surgical dissection of the left kidney. (b) Wedge resection partial nephrectomy performed on the lower pole.

Source: Elaborated by the authors.

Following a single dose of broad-spectrum antibiotic administration, general anesthesia was induced via intraperitoneal injection of ketamine (50 mg/kg) and xylazine (5 mg/kg). A midline laparotomy was performed following sterile preparation, allowing dissection to proceed to the left kidney, which was completely mobilized with minimal manipulation of the surrounding tissues. Partial nephrectomy was performed on lower pole through wedge resection, without application of manual pressure or hilar vascular control, at resection angle of 90° and depth of 3 mm (Fig. 1).

**Figure 2:**
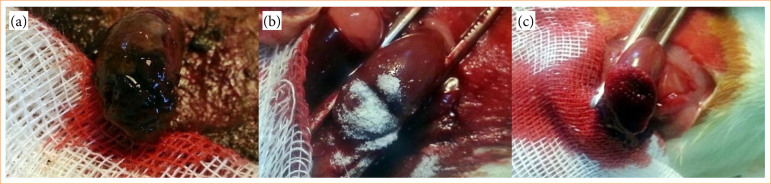
Application of hemostatic agents after partial nephrectomy. (a) Ankaferd; (b) Arista; (c) Surgiflo.

Source: Elaborated by the authors.

Blood was collected from edge of bleeding surface in Ankaferd, Arista, and Surgiflo groups, while mild compression with sponge was applied to bleeding surface in control group; all sponges pre-weighed on sensitive scale. In the treatment groups, a single application was administered to cover the bleeding surface, using 0.2 mL of Ankaferd, 0.3 g of Arista, or 0.8 mL of Surgiflo, each corresponding to one-tenth of the standard product dose (Fig. 2). All animals survived throughout the surgical procedure and the postoperative period.

**Figure 3:**
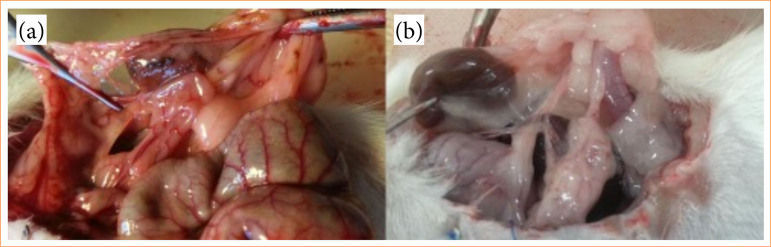
Assessment of intra-abdominal adhesions. (a) Limited vascular adhesions separating with traction. (b) Dense adhesions separating by sharp dissection.

Source: Elaborated by the authors.

On the tenth day after the initial surgery, all rats were anesthetized via intraperitoneal injection of ketamine (50 mg/kg) and xylazine (5 mg/kg), followed by a laparotomy to evaluate intra-abdominal adhesions and harvest the left kidneys for histopathological analysis by a blinded surgeon. The degree of adhesions was classified as follows:

0: no adhesion;
1: avascular adhesions separating spontaneously;
2: limited vascular adhesions separating with traction;
3: dense adhesions separating by sharp dissection^8^ (Fig. 3).

All surgical procedures and product applications were conducted by the same surgeon (IU).

**Figure 4:**
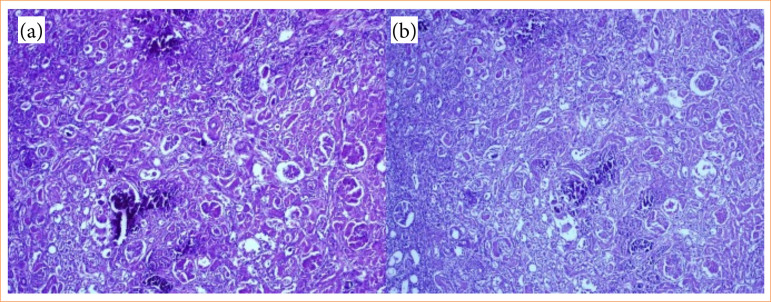
Histopathological changes: (a) glomerular necrosis in Ankaferd group (hematoxylin and eosin, x10); (b) tubular necrosis in Surgiflo group (hematoxylin and eosin, x10).

Source: Elaborated by the authors.

Nephrectomy samples were fixed in 10% formalin and embedded in paraffin. Four-μm sections were stained with hematoxylin and eosin (H&E) and examined under a light microscope to assess histopathological changes in tissues adjacent to the resection site. The degree of tubular and glomerular necrosis was defined as follows:

0: no injury;
1: injury in 1–10% of tubules/glomeruli;
2: injury in 11–25% of tubules/glomeruli;
3: injury in 26–75% of tubules/glomeruli;
4: injury in 76–100% of tubules/glomeruli (Fig. 4).

For immunohistochemical analysis, 4-μm sections were placed on L-lysine coated slides and stained with a CD142 antibody, with both cytoplasmic and nuclear staining considered positive. The degree of CD142 staining was defined as follows:

0: no staining;
1: staining in 1–10% of tubules;
2: staining in 11–25% of tubules;
3: staining in 26–75% of tubules;
4: staining in 76–100% of tubules.

### Statistical analysis

The Statistical Package for the Social Sciences (IBM Corp., version 29.0) software package was used in the analysis of data. Descriptive statistics were reported as medians and interquartile ranges (IQR). Normality of the amount of bleeding and bleeding time data was assessed using the Shapiro-Wilk test. For ordinal variables and continuous variables that did not follow a normal distribution, intergroup differences were analyzed using the Kruskal-Wallis test. When a significant difference was detected, pairwise comparisons were conducted using Dunn’s post-hoc test with Bonferroni correction. *P* < 0.05 was defined as the level of statistical significance.

## Results

The total amount of bleeding was the lowest in the Ankaferd group (0.33 [0.30–0.44] g), showing a statistically significant reduction compared with the control group (1.97 [1.75–2.09] g) (*p* < 0.001). A significant difference was also observed between the Surgiflo (0.67 [0.57–0.88] g) and control groups (*p* = 0.038), whereas no other pairwise comparisons reached statistical significance. Bleeding time was the shortest in the Ankaferd group (17 [14–23] s), followed by the Arista (35 [33.5–36.5] s), Surgiflo (37 [34–41] s), and control (180 [175–185] s) groups. The Ankaferd (*p* < 0.001) and Arista (*p* = 0.046) groups demonstrated significantly shorter bleeding times compared with the control group. No significant differences were observed among the other groups in terms of bleeding time (Table 1).

**Table 1:** Comparison of amount of bleeding and bleeding time among groups.

	Amount of bleeding, g (median—IQR)	*p* -value	Bleeding time, s (median—IQR)	*p* -value
Control	1.97 (1.75–2.09)	< 0.001* *versus* Ankaferd 0.192 *versus* Arista 0.038* *versus* Surgiflo	180 (175–185)	< 0.001* *versus* Ankaferd 0.046* *versus* Arista 0.149 *versus* Surgiflo
Ankaferd	0.33 (0.30–0.44)	0.068 *versus* Arista 0.307 *versus* Surgiflo	17 (14–23)	.283 *versus* Arista 0.097 *versus* Surgiflo
Arista	0.89 (0.82–1.15)	1.000 *versus* Surgiflo	35 (33.5–36.5)	1.000 *versus* Surgiflo
Surgiflo	0.67 (0.57–0.88)	-	37 (34–41)	-

IQR: interquartile range;

* Kruskal-Wallis test with Dunn-Bonferroni post-hoc correction. Source: Elaborated by the authors.

In the analysis of adhesion score distribution, the Ankaferd group was significantly more frequently represented in the higher adhesion score categories compared with the control group (*p* = 0.01). No statistically significant differences in adhesion score distribution were observed among the other groups (Table 2). The glomerular necrosis score was significantly higher in the Ankaferd group compared with the control group (*p* = 0.043), while the tubular necrosis score did not differ significantly among the groups. Similarly, no statistically significant differences were observed among the groups in the CD142 immunohistochemical evaluation.

**Table 2:** Comparison of adhesion score distribution among groups.

	Adhesion score (median—IQR)	*p* -value
Control	1 (0–1)	0.010* *versus* Ankaferd 1.000 *versus* Arista 1.000 *versus* Surgiflo
Ankaferd	2 (2–3)	0.059 *versus* Arista 0.137 *versus* Surgiflo
Arista	1 (0–1.5)	1.000 *versus* Surgiflo
Surgiflo	1 (0.5–1.5)	-

IQR: interquartile range; *

* Kruskal-Wallis test with Dunn-Bonferroni post-hoc correction Source: Elaborated by the authors.

## Discussion

Bergel was the first to use hemostatic agents for achieving hemostasis in partial nephrectomy, after which various animal studies and clinical trials were reported in the literature^9^. Before incorporating this wide variety of agents into clinical practice, their hemostatic efficacy, potential to cause allergic reactions or transmit viruses, nephrotoxic components, and possible carcinogenicity should be elucidated through comparative animal studies. To date, no studies have comparatively evaluated the hemostatic efficacy and safety profiles of Ankaferd, Arista, and Surgiflo in a partial nephrectomy model, apart from the present study.

The mechanism of action of Ankaferd involves the formation of an encapsulated protein network in less than 1 second, which provides focal points for vital erythrocyte aggregation^10^. Huri et al. analyzed the hemostatic efficacy and histopathological features of Ankaferd in a rat partial nephrectomy model^11^. A total of 28 rats were divided into four groups:

1. Hilar control + parenchymal suture;
2. Hilar control + parenchymal suture + Ankaferd;
3. Hilar control + Ankaferd;
4. Ankaferd.

Warm ischemia time (*p* = 0.011) and operation time (*p* = 0.007) were significantly shorter in group 3 compared to group 1. While group 1 exhibited significantly increased glomerular necrosis and calcification, the Ankaferd-related groups showed increased erythrocyte aggregation, which was reported to be associated with the mechanism of action.

One of the most important parameters in determining the safety of hemostatic agents for intra-abdominal use is the degree of adhesion formation. Tokgöz et al.^8^ did not identify a significant difference between the sham, control, and Ankaferd groups in a rat partial nephrectomy model. Similarly, Karaca et al.^12^ found no difference between the saline, control, and Ankaferd groups in a rat peritoneal adhesion model with respect to intra-abdominal adhesions. In contrast to these studies, we observed significantly more pronounced intra-abdominal adhesions in the Ankaferd group compared to the control group.

There are no studies evaluating the hemostatic efficacy of Surgiflo in partial nephrectomy animal models, but Slezak et al.^13^ compared Surgiflo to another gelatin matrix agent, Floseal (Baxter), in a porcine kidney trauma model. At the 2-minute time point after application of the hemostatic agents, lesions treated with Floseal exhibited significantly less bleeding than those treated with Surgiflo (*p* < 0.001). This difference remained significant even for lesions with an initial bleeding rate of ≥ 29 mL/min (*p* = 0.04).

There are limited studies in the literature regarding the efficacy and safety of Arista in partial nephrectomy. Murat et al.^14^ evaluated Arista and oxidized regenerated cellulose Surgicel (Ethicon) in a porcine partial nephrectomy model. Both ischemia and hemostasis times were significantly reduced in the Arista group (*p* = 0.004), while no significant difference was observed in blood loss. More notably, a clinical trial conducted by Palacios et al.^15^ revealed no significant differences between Arista and Floseal in terms of blood loss, warm ischemia time, and postoperative complications in patients undergoing partial nephrectomy. Hoffmann et al.^16^ evaluated several hemostatic agents, including Arista, in a rat cecal adhesion model, and observed that the Arista group had a significantly lower adhesion score (3.9 *versus* 6, *p* < 0.05) compared to the control group. This finding aligns with our study, which demonstrated no significant difference in adhesion score distribution between the Arista and control groups.

In our study, as a primary endpoint, Ankaferd demonstrated superior performance compared to Arista and Surgiflo in terms of both amount of bleeding and bleeding time. The superiority of Ankaferd in hemostasis was overshadowed by its higher mean adhesion and glomerular necrosis scores, which may weaken its safety profile. CD142, which plays a key role in the coagulation cascade upon activation by vascular damage, did not differ between the study groups. For more reliable clinical application, further studies should focus on zero-ischemia partial nephrectomy models to compare the hemostatic efficacy of these agents^17^.

The limitations of our study include the lack of body temperature stabilization and the absence of evaluation of anesthetic agents, both of which may affect bleeding parameters. In addition, the estrous cycles of the female rats were not monitored, which might have contributed to physiological variability. Another limitation is the absence of preoperative and postoperative hemoglobin measurements, which could better reflect the clinical bleeding levels. Further studies may assess the compatibility of histopathological changes induced by these agents with human physiology, as well as investigate their absorption processes and the metabolic effects following degradation.

## Conclusion

Ankaferd achieved hemostasis more rapidly and effectively compared to Arista and Surgiflo. The increased adhesions observed in the Ankaferd group compared to the control group reduced its suitability for intra-abdominal use. The identification of ideal hemostatic agents, along with a better understanding of their mechanisms of action and metabolic effects, requires further standardized, comparative, and prospective clinical trials.

## Data availability statement

The data will be available upon request.
